# The effect of a self-care application on the quality of life of women with pelvic organ prolapse: a randomized controlled trial

**DOI:** 10.1186/s12905-025-04241-w

**Published:** 2025-12-29

**Authors:** Zohra Mousavi, Zahra Hadizadeh-TalaSaz, Taraneh Mohajeri, Jamshid Jamali

**Affiliations:** 1 Department of Midwifery, School of Nursing and Midwifery, Mashhad University of Medical Sciences, Mashhad, Iran; 2 Nursing and Midwifery Care Research Center, Mashhad University of Medical Sciences, Mashhad, Iran; 3 Department of Obstetrics and Gynecology, Faculty of Mashhad Medical Sciences, Islamic Azad University, Mashhad, Iran; 4 Department of Biostatistics, School of Public Health, Mashhad University of Medical Sciences, Mashhad, Iran; 5 Social Determinants of Health Research Center, Mashhad University of Medical Sciences, Mashhad, Iran

**Keywords:** Pelvic organ prolapse, Application, Self-care, Quality of life, Mobile application

## Abstract

**Background:**

Pelvic organ prolapse (POP) is a public health problem that affects individuals’ psychosocial quality of life (QOL). However, affected women are often hesitant to seek care and treatment. Access to treatment and self-management for this condition can be supported through self-care applications.

**Objective:**

This study aimed to determine the effect of a self-care application on the quality of life of women with pelvic organ prolapse.

**Methods:**

This RCT was conducted at Imam Reza Hospital and private women’s clinics in Mashhad, Iran, from February 2024 to August 2024. 64 women were randomly assigned to either the application group or the control group via block randomization, and 56 women completed the study. The control group received pamphlets and face-to-face education, whereas the intervention group used self-care applications alongside routine education. The participants completed a demographic questionnaire and the Pelvic Organ Prolapse Quality of Life questionnaire (P-QOL) before and immediately after two months of intervention. A lower score reflects a higher quality of life. For the secondary outcome, participants’ knowledge was assessed before and after the intervention using the Prolapse and Incontinence Knowledge Questionnaire (PIKQ). In addition, the prolapse stage was assessed by the Pelvic Organ Prolapse Quantification (POP-Q). The data were analyzed via SPSS Version 16.

**Results:**

Before the intervention, the median quality-of-life score in the intervention group and in the control group was not statistically significant (*P* = 0.501). However, the median change in quality of life scores from pre- to post-intervention was − 14.58 (IQR:-20.73 to -11.14) in the intervention group and − 7.49 (IQR:-11.10 to -3.34) in the control group (*P* < 0.001). After intervention, the proportion of participants with an improvement of one or more POP-Q stages showed no difference between the groups (*P* = 0.880). After the intervention, the median knowledge score in the intervention group was significantly higher than the control group (*P* < 0.001).

**Conclusion:**

The application was effective in improving the quality of life of women with symptomatic stage I-II pelvic organ prolapse. In situations where self-care is appropriate, a self-care application can be a suitable alternative to other treatments or other conservative management approaches.

**Trial registration:**

This trial was registered in the Iranian clinical trials database (irct.behdasht.gov.ir) under the code IRCT20230923059497N1. Date of first registration was: 02/12/2023.

## Introduction

The International Continence Society and the International Urogynaecology Association define POP as the descent of one or more pelvic organs (such as the uterus or the vaginal apex, bladder, or rectum) from their normal anatomical positions into the vagina [1]. Pelvic organ prolapse (POP) can occur due to the weakening and laxity of the muscles and ligaments that hold the pelvic organs in place, usually stemming from factors such as childbirth, the aging process, chronic coughing, obesity, or previous pelvic surgery [2]. POP is associated with multiple symptoms that can affect multiple aspects of women’s lives, such as bowel, lower urinary tract, and sexual dysfunction [3]. POP is not life-threatening, but it is one of the main causes of disability among women and has a significant effect on their quality of life [4]. Hadizadeh-Talasaz et al., in their systematic review and meta-analysis, reported that the overall prevalence of POP was 30.9%, depending on the research methods used. When symptoms were assessed by a questionnaire, the estimated prevalence was 25.0%, whereas when symptoms were assessed by a clinical examination (Pelvic Organ Prolapse Quantification), the prevalence was 41.8% [5]. POP is a reason for consultation, pelvic physiotherapy, medication, specialist care, and surgical interventions, which impose high costs on women and society [6]. Conservative methods and behavioral modifications are considered the first line of treatment for this condition, even in severe cases [7, 8]. Pelvic organ prolapse is a chronic condition that requires long-term self-care. Patient participation in the treatment process contributes to better outcomes and can improve women’s quality of life. However, programs related to pelvic floor disorders are sometimes challenging because they require multiple appointments and the involvement of specialists, so many women prefer to decline services [9]. In today’s modern world, the difficulty of frequent in-person visits can be reduced by new technology. Smartphone technology has provided a new field for patient self-care. With the increasing availability of smartphones, mobile health apps are growing and offering new opportunities for the delivery of healthcare services. Health apps can improve easy access to healthcare for people who are unwilling or unable to seek care. It is believed that health apps can increase adherence to disease treatment [10]. Currently, various apps have been developed to assist in the treatment of different diseases and have shown satisfactory results [11–13]. Waddensten et al. reported that the use of a self-management app for the treatment of urge incontinence and urinary incontinence increased access to first-line treatment and significantly improved quality of life and symptom severity [14]. To our knowledge, there is no app for people with POP in the Persian language; therefore, we developed a mobile app with a treatment program focused on pelvic floor exercises and behavioral modifications. Given the high prevalence of POP, the important role of self-care in improving symptoms, and the widespread availability of mobile phones, the present study aimed to determine the effect of a self-care app on the quality of life of women with POP.

## Method

This research is a two-arm randomized clinical trial (RCT) with a pre- and posttest design conducted at two pelvic floor disease specialty clinics in Mashhad (Iran) from February 2024 to August 2024. This trial was registered in the Iranian clinical trials database at (irct.behdasht.gov.ir) under the code IRCT20230923059497N1 on 02/12/2023. There were no changes to the trial design, outcomes, or analyses after the study began. We followed the CONSORT guideline. Sixty-four women with symptomatic POP who were referred to the gynecology clinic of Imam Reza Hospital and private women’s clinics in Mashhad and met the inclusion criteria were enrolled in the study. The inclusion criteria included symptoms of pelvic organ prolapse (based on screening questions), physician confirmation of prolapse stage II or higher according to the Pelvic Organ Prolapse Quantification (POP-Q) system, Women with mild urinary incontinence, determined according to scores on the International Consultation on Incontinence Questionnaire–Urinary Incontinence Short Form )ICIQ-UI SF score 1–5(, being married, being literate (able to read and write), owning a smartphone or tablet with the Android operating system, being able to use a smartphone, having no history of pelvic surgery in the past year, having no history of pelvic physiotherapy in the past year, and not given birth in the past year. The exclusion criteria included unwillingness to continue participation, discontinuation of communication with the researcher for more than one week, use of another type of treatment during the study, and pregnancy.

### Sampling, randomization, and masking

Sampling was initially conducted using a convenience sampling method, and then participants were assigned to either the intervention or control group through a randomized allocation method. Permuted block randomization was used to generate the allocation sequence of individuals to the study groups. This study involved six blocks: (1) AABB (2), ABAB (3), ABBA (4), BBAA (5), BABA, and (6) BAAB. The sequence of random allocation of individuals was generated via Allocation Random Software, with a block size of four. Due to the nature of the intervention and self-reported outcomes, neither the participants nor the researcher collected data was blinded to the intervention. However, the physician conducting the examinations remained blinded to group allocation throughout the entire study period.

### Sample size

To estimate the sample size, a formula for comparing means with standard deviation in two populations was used. Based on Wadensten et al., [14] who reported post-intervention quality of life (QoL) scores with means and standard deviations of 8.7 ± 8.29 in the intervention group and 0.9 ± 5.36 in the control group, and considering a 5% significance level (alpha = 0.05) and 80% power (beta = 0.20), the sample size for comparing two independent means was determined to be a minimum of 26 participants per group. Given the interventional nature of the study and an anticipated dropout rate of about 20%, the final sample size was set at 32 participants per group.

$$\begin{aligned}\:\boldsymbol{n}&=\frac{{\left({\boldsymbol{z}}_{1-\frac{\boldsymbol{\alpha\:}}{2}}+{\boldsymbol{z}}_{1-\boldsymbol{\beta\:}}\right)}^{2}\left({\boldsymbol{s}}_{1}^{2}+{\boldsymbol{s}}_{2}^{2}\right)}{{({\stackrel{-}{\boldsymbol{x}}}_{2}-{\stackrel{-}{\boldsymbol{x}}}_{1})}^{2}}\\&=\frac{{\left(1.96+0.84\right)}^{2}\left({7.8}^{2}+{9}^{2}\right)}{{(29.8-36.5)}^{2}}=26\end{aligned}$$

### Ethical consideration

To adhere to ethical considerations, the necessary explanations about the research, its implementation, and the confidentiality of collected information were provided to potential participants who met the inclusion criteria. After obtaining informed consent, participants were recruited into the study.

### Instruments

At the beginning of the study, the ICIQ-UI SF questionnaire was completed by participants to exclude individuals with moderate to severe urinary incontinence. This questionnaire consists of three questions asking about the frequency, amount, and impact on everyday life. Score range was (0–21), which can be further categorized into four categories (1–5 points = mild, 6–12 points = moderate, 13–18 points = severe, 19–21 points = very severe) [15].

Participants enrolled in the study initially completed the demographic and obstetric information questionnaire. The accuracy of its contents was checked by seven experts from members of the scientific board of Mashhad University of Medical Sciences.

The Pelvic Organ Prolapse Quality of Life (P-QOL) questionnaire was completed by both groups at the beginning of the study and immediately after two months of intervention. The P-QOL questionnaire is a self-report instrument with 38 questions. The 20 questions represent 9 areas of quality of life, including general health (1 item), prolapse impact (1 item), role limitations (2 items), physical limitations (2 items), social limitations (3 items), personal relationships (2 items), emotions (3 items), sleep/energy (2 items), and intensity measurement (4) items. Scores in each domain are calculated via the formula provided by the author of the original article and range from 0 to 100. A lower score indicates a better quality of life in each specific domain. In addition, there are 18 questions about urinary, bowel, and vaginal prolapse symptoms that do not have specific scores [3]. For scoring, max–min normalization was initially used, and the scores of each dimension were placed in the range of 0- 100. In this study, the reliability of the quality-of-life dimensions, as measured by Cronbach’s alpha, ranged from 0.67 to 0.82.

In the POP-Q system, the degree of prolapse of the vaginal wall or cervix is assessed using the hymen as a reference point, with the patient in the lithotomy position and performing the Valsalva maneuver at the beginning of the study and immediately after two months of intervention. POP-Q staging was performed by a physician fellowship-trained in pelvic floor disorders, who was blinded to group allocation throughout the study. First author assisted during the examinations, while the physician made all final staging decisions.

The Pelvic Organ Prolapse and Urinary Incontinence Knowledge Questionnaire (PIKQ) was used as the standard tool for assessing participants’ knowledge. Participants responded to the items with the options “Agree,” “Disagree,” or “Don’t know.” Correct answers were scored as 1, while incorrect or “Don’t know” responses were scored as 0. The total score for each scale ranged from 0 to 24, with higher scores indicating greater knowledge about urinary incontinence and/or pelvic organ prolapse. This questionnaire assesses three domains of knowledge related to etiology, diagnosis, and treatment. The reliability and validity of this tool were assessed and approved in a previous study [16].

### Intervention group

The intervention was a Persian-language mobile application made for Android devices. Our research group developed this app in collaboration with a software engineer. During the development process, the app was reviewed by the researchers and a test group until a final version was accepted. During the study period, no changes were made to the app. The main features of the application include a user profile, reminders, daily statistics, educational content, search functionality, the ability to share the app via platforms such as WhatsApp and Eitaa, a timer for performing Kegel exercises, and an option to contact a health professional. The app focused on pelvic floor exercises but also contained information that described prolapse. The application was structured into nine separate menus: (1) information about pelvic organ prolapse (definition, symptoms, types, causes, risk factors, diagnosis, prevention, and treatment); (2) self-care recommendations (weight management, nutritional advice, vitamin D and C intake, constipation prevention, the Knack maneuver, pelvic floor muscle exercises, sexual health advice, and stress and anxiety management); (3) a structured Kegel exercise program; (4) exercise demonstrations presented through videos and written instructions, supported by a built-in timer for contractions and relaxation periods and recording daily exercise statistics; (5) content search functionality; (6) instructional videos on application use; (7) information about the research team; (8) user account login; and (9) communication with the healthcare provider.

The description of each exercise included a video recorded by the researcher, which showed the correct technique to perform the exercises, the intensity, and the duration of contraction and relaxation. The exercises included different combinations of contractions, such as: Simple contractions to identify the correct muscles, exercises in various positions (Bridge, Squat, Standing) to improve strength, quick contractions, and contractions preceding coughing (knack maneuver). Participants were instructed to perform the exercises in three sets of 10 repetitions for each exercise throughout the day at least five days a week. Participants received app reminders three times per day to encourage engagement in the exercise routine. After completing each exercise, women could save their performance in a statistical table. The goal was regular exercise for two months, not reaching a specific level of fitness. Also, Participants were instructed to read or listen to the educational materials at least once. Then, changes in knowledge were measured using a structured questionnaire. The content was developed based on an extensive review of relevant literature and articles from reputable evidence-based resources (UpToDate, ClinicalKey, Cochrane) and major scientific databases (Ovid, PubMed, Embase), as well as expert consultation. After gathering information on self-care for individuals with prolapse, the content was validated by seven experts. The educational content was designed using simple, non-technical language suitable for the general population, using a mixed-format approach, including text, audio, and video content. All participants in the intervention group received identical, standardized content. The total time required to review all educational materials, including videos, was approximately one hour.

The intervention group, in addition to the routine visit and receiving a routine educational pamphlet, received instructions on how to install and use the application during a 30–60 min individual training session after their visit. Participants received a personal account along with guidance to download and install the app on their tablet or smartphone. An Eitaa group (an Iranian application for chat) was formed to answer the participants’ questions. No participants reported any adverse effects of pelvic floor muscle training.

App usage: We monitored app usage through three types of data: (1) participants regularly took screenshots of their usage statistics and sent them to the researcher via the Eitaa messaging platform, and (2) each time a participant logged into the app, a notification was automatically generated for the researcher, indicating active app engagement (3). Changes in participants’ knowledge were assessed using the Pelvic Organ Prolapse and Urinary Incontinence Questionnaire (PIKQ) before and after the intervention.

### Control group

The control group received educational content through face-to-face sessions and printed pamphlets. This group did not receive the app or any material included in the app during the study period. After completing the 2-month follow-up, they received access to the app.

### Outcomes

At baseline, participant demographic characteristics and obstetric information were collected. Primary and secondary outcomes were measured at baseline and immediately after two months of intervention. For the primary outcome, we consider quality of life, assessed using the P-QOL questionnaire. The primary analysis was conducted based on between-group comparisons of post-intervention P-QOL scores. To complement the primary analysis, within-group comparisons were also performed to assess changes in quality of life from pre- to post-intervention. Comparison of P-QOL domains in the two groups was also analyzed as components of the primary quality-of-life outcome. For the secondary outcome, participants` knowledge was assessed before and after the intervention by PIKQ. At the beginning and the end of the second month, an examination was performed for both groups in the physician’s office to assess changes in the degree of prolapse by the POP-Q system.

### Statistical methods

The data analysis for statistical purposes was done via SPSS software version 16. Descriptive statistics were calculated to describe the Baseline data (means and standard deviation for continuous variables and numbers and percentages for categorical variables). The Shapiro‒Wilk test was used to examine the normality of the distribution of quantitative variables. The results of the Shapiro‒Wilk test revealed that the variables of body mass index and number of pregnancies in the two control and intervention groups followed a normal distribution. In contrast, the other quantitative variables did not follow a normal distribution in at least one of the two intervention and control groups. Therefore, to examine the homogeneity of the variables of body mass index and number of pregnancies in the two groups, the independent parametric t-test was used, and in other cases, the nonparametric Mann‒Whitney test was used. Categorical variables were analyzed using either the Chi-square test or the Fisher’s exact test, depending on the data distribution. The Chi-square test was used when the assumptions regarding the expected cell counts were met, whereas Fisher’s exact test was used for contingency tables with small expected frequencies (i.e., < 5) in any cell.

The Mann‒Whitney test was used to compare the quality of life between two groups before intervention due to non-normal distribution. After the intervention, the quality-of-life scores were normally distributed; therefore, between-group comparisons were performed using the independent t-test. The changes in scores from pre- to post-intervention in total score and all dimensions of quality of life were not normally distributed in both groups. Therefore, the Mann–Whitney U test was used for between-group comparisons. For intragroup comparisons, paired t-tests and Wilcoxon tests were used based on data distribution.

The PIKQ was analyzed using the same statistical approach as quality of life results, with the choice of parametric or non-parametric tests based on the distribution of the data. All statistical analyses were done using a two-sided significance level, with a predefined alpha of 0.05.

## Results

**Fig. 1:**
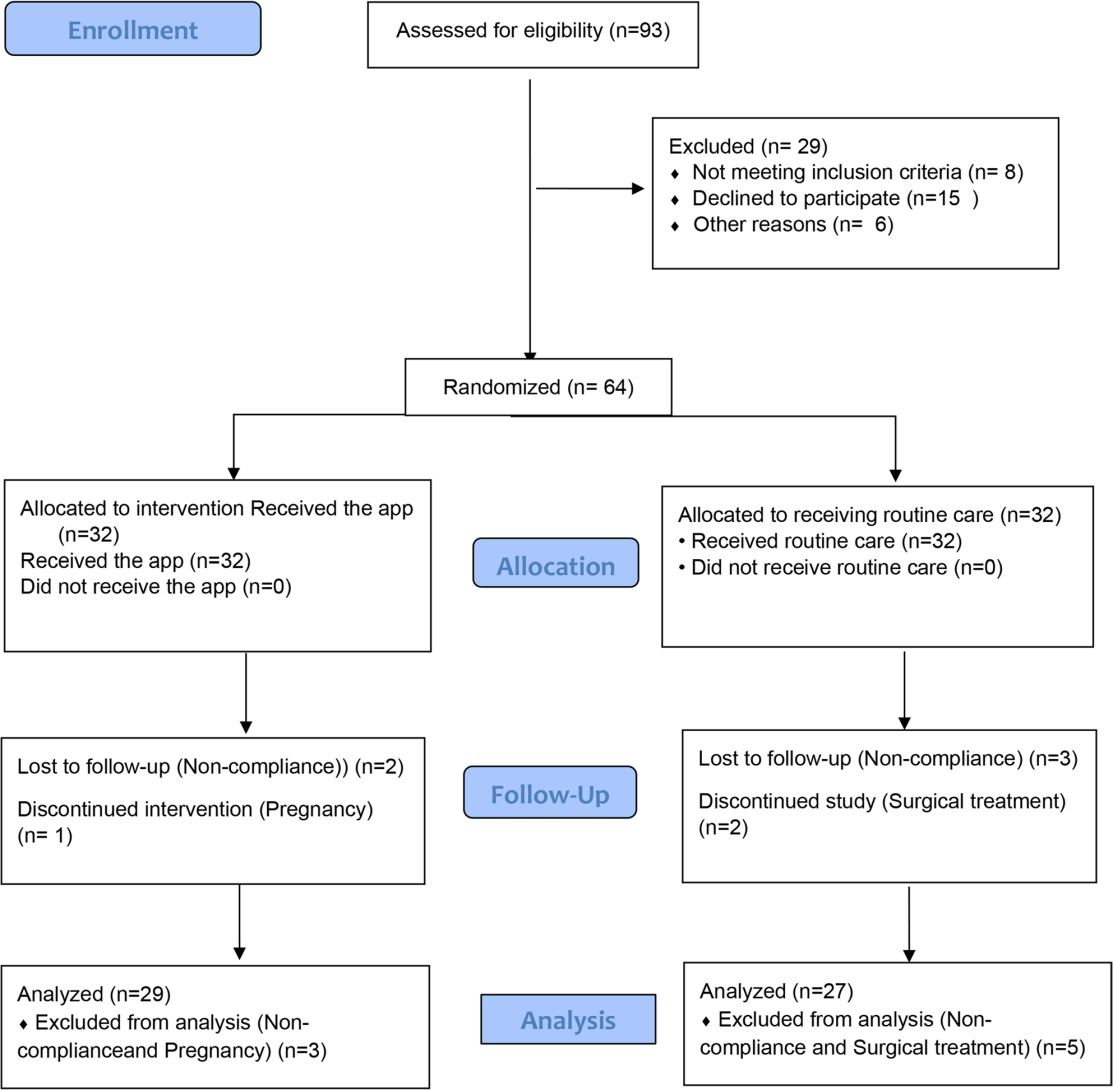
CONSORT flow diagram

Of the 93 assessed for eligibility, 64 were eligible and allocated randomly into two groups evenly. At the end of the study, 8 participants were excluded (3 in the intervention group, one due to pregnancy, two due to weak cooperation to perform exercises and use app consistently in the study, and 5 in the control group due to weak cooperation to perform exercises and use app consistently and the use of surgical treatment). Finally, 29 participants in the intervention group and 27 participants in the control group remained and were subjected to statistical analysis. The final analysis was conducted on 56 participants (Fig. 1).

The personal information of the subjects in the two intervention and control groups was compared in Table 1. The results of the statistical tests revealed that the demographic and obstetric variables of both intervention and control groups were homogeneous and consistent (Table 1).

**Table 1:** Comparison of demographic and obstetric variables between the intervention and control groups

Variable	Group			Classification	Test result
	Control (27 people)	Intervention (29 people)			
	Number(percentage)	Number (percentage)			
Prolapse Stage	9(33.3)	7(24.1)		I	F = 1.764 *p* = 0.461
	18(66.7)	22(75.9)		II	
	27(100.0)	29(100.0)		Total	
Patient’s education level	3(11.1)	3(10.3)		elementary	F = 2.567 *P* = 0.512
	3(11.1)	2(6.9)		middle school	
	6(22.2)	12(41.4)		high school	
	15(55.6)	12(41.4)		Academic	
Job	13(52.0)	20(69.0)		Housewife	F = 2.903 *P* = 0.161
	12(48.0)	8(27.6)		Employed	
	0(0.0)	1(3.4)		Retired	
Type of delivery	19(73.1)	22(78.6)		Natural childbirth	F = 0.609 *P* = 0.892
	5(19.2)	5(17.9)		Cesarean section	
	2(7.7)	1(3.6)		Both	
Episiotomy history	8(29.6)	12(41.4)		No	χ^2^ = 0.841 *P* = 0.412
	19(70.4)	17(58.6)		Yes	
Chronic cough	22(88.0)	25(89.3)		No	*P* ≈ 1.00 F = 0.022
	3(12/0)	3(10/7)		Yes	
Chronic constipation	15(57.7)	2(69.0)		No	χ^2^ = 0.753 *P* = 0.415
	11(42.3)	9(31.0)		Yes	
Smoking	25(96.2)	29(100.0)		No	F = 1.136 *P* = 0.473
	1(3.8)	0(0.0)		Yes	
Family history of prolapse	16 (69.6)	17 (73.9)		No	χ^2^ = 0.107 *P* = 0.982
	7 (30.4)	6 (26.1)		Yes	
				χ^2^ = Κ−2 test	F = Fisher’s exact test
		Mean± SD			
Age		10.5 ± 39.03	8.41 ± 39.22	*P* = 0.786	z = 0.271
Body mass index		3.42 ± 27.34	1.97 ± 26.92	*P* = 0.571	t = 0.570
Baby weight		0.52 ± 3.27	0.40 ± 3.46	*P* = 0.244	1.661-z=
Number of pregnancies		1.38 ± 2.76	1.34 ± 2.89	*P* = 0.722	t = 0.358
Number of births		1.21 ± 2.38	1.07 ± 2.30	*P* = 0.925	z = 0.094
Age at first birth		3.89 ± 22.70	4.37 ± 23.36	*P* = 0.434	z = 0.783

The results of the primary outcome revealed that, before the intervention, the median quality of life score in the intervention group was 29.57 (IQR: 24.58–44.44) and in the control group, the median score was 39.16 (IQR: 24.58–49.99), which was not statistically significant (*p* = 0.501). After the intervention, the median quality-of-life score in the intervention group was 16.65 (IQR: 9.84–27.21), in the control group, the median score was 29.61 (IQR: 21.23–40.73), with a significant difference observed between the groups (*p* = 0.002). The median change in quality of life scores from pre- to post-intervention was − 14.58 (IQR: -20.73 to -11.14) in the intervention group and − 7.49 (IQR: -11.10 to -3.34) in the control group. The difference between groups was statistically significant (Mann–Whitney U test, *p* < 0.001). In the intragroup comparison, the result revealed a significant difference between the stages in the intervention and control group (*p* < 0.001) (Table 2).

**Table 2:** Median (IQR) Quality-of-Life scores before and after the intervention and their statistical comparisons between the two groups

Quality of life score	Group		Intergroup test result
	Intervention (29 people)	Control (27 people)	
	Median (third quartile, first quartile)	Median (third quartile, first quartile)	
Before the intervention	29.57(24.58,44.44)	39.16(24.58,49.99)	*P* = 0.501 Z = 0.67
After the intervention	16.65(9.84,27.21)	29.61(21.23,40.73)	t = *-3.28 = *P* 0.002
Changes after intervention to before intervention	14.58- (11.14- ,20.73-)	7.49- (3.34-, 11.10-)	*P* **>** 0.001 Z = 4.51*
Intragroup test result	* t=-13.30	z =-3.77*	
	*P* > 0.001	*P* > 0.001	
	Dependent t test	Wilcoxon test	

*Significant at the 5% level

The results of the study examining the effect of the self-care application on the dimensions of quality of life in women with (POP) revealed that the median change after the intervention in role limitations (*p* = 0.036), emotions (*p* < 0.001), Sleep/Energy (*p* = 0.049), and Severity Measurement (*p* = 0.019), indicating a significant difference between the two groups. (Table 3).

**Table 3:** Median (IQR) of changes in scores before and after intervention in quality-of-life dimensions

Dimensions of quality of life	Group		Test result (Mann‒Whitney test)
	Intervention (29 people)	Control (27 people)	
	Median (third quartile, first quartile)	Median (third quartile, first quartile)	
General Health	0.0(0.0,0.0)	0.0(-25,0.0)	z = 0.043
			*P* = 0.101
Impact of Prolapse	0.0(-33.30,0.0)	0.0(-33.30,0.0)	z = 0.781
			*P* = 0.830
Role Limitations	-16.65(-16.70,0.0)	16.70(-33.35, -16.65)	= z *2.096
			*P* = 0.036
Physical Limitations	-16.70(-16.70, 0.07)	-16.70(-33.32, -8.32)	z = 0.874
			*P* = 0.382
Social Limitations	0.0(-16.65, 0.0)	0.0(-16.65, 0.0)	z = 1.849
			*P* = 0.064
Personal Relationships	0.0(0.0, 0.0)	0.0(-11.3, 0.0)	z = 1.890
			*P* = 0.059
Emotions	-11.10(-11.10, 0.0)	-22.20(-23.35.-11.10)	= z *3.850
			0.001 **>** P
Sleep/Energy	16.65(-16/70, 0.0)	-16.70(-33.35, 16.65)	= z *1.966
			*P* = 0.049
Measurement Severity	0.0(-8.32, 0.0)	-8.32(-16.66, 0.0)	= z *2.346
			*P* = 0.019

*Significant at the 5% level

The results of the secondary outcome revealed that, after intervention, the proportion of participants with an improvement of one or more POP-Q stages showed no difference between the groups (*P* = 0.880).

Another secondary outcome revealed that, before the intervention, the median pelvic organ prolapse–related knowledge score did not differ significantly between the two groups (*p* = 0.740). However, after the intervention, the median knowledge score in the intervention group in all domains was significantly higher than that of the control group (*p* < 0.001), and the within-group comparison indicated a significant improvement only in the intervention group (*p* = 0.016).

## Discussion

The results of this clinical trial revealed that using two-month of selfcare mobile application on POP, compared with routine treatment, resulted in a statistically significant improvement in the quality of life of women with stage I-II pelvic organ prolapse. A study by Due et al. showed that lifestyle recommendations alone can improve quality of life, and combining them with pelvic floor exercises increases their effect in women with POP [17]. In the same vein, we combined exercises and educational content related to pelvic organ prolapse in the application. Aligned with our findings, Wadensten et al. also revealed that a self-management application for women with urgency and mixed incontinence is effective for improving the quality of life, not only for short-term follow-up but also for long-term follow-up [18]. Asklund et al. mentioned in their qualitative study that participants were grateful for providing a new and modern way to access a treatment. The participants stated that new technology-assisted care reduced feelings of embarrassment and helped them to take responsibility for their treatment. Additionally, it motivates women to perform pelvic floor exercise [19]. Recent studies have shown that E-health interventions are effective for the treatment and prevention of pelvic floor disorders. One possible way to meet the needs of the medical industry in the future is to increase the self-management capabilities of patients through applications. A systematic review has shown that self-care applications improve health outcomes [20]. The results of recent studies have shown that self-care applications are convenient, flexible, accessible, and save time, which are potentially effective at providing PFMT to women [19]. Similarly, a systematic review by [20] revealed that, compared with the control group, the application group presented significant improvements in the severity of stress urinary incontinence symptoms, quality of life, and perceived global improvement. In addition, this group showed a significant improvement in PFMT adherence. The results of this review indicated that application-based PFMT is promising from the perspective of improving outcomes and adherence to exercise, which was consistent with the results of the current study [20]. As mentioned in other studies, pelvic floor exercises can play a role in improving women’s quality of life [15], and given that most people have barriers to in-person treatment of this disease, including shame, lack insurance coverage, and lack of awareness, using an educational app can partially eliminate these barriers and help women better improve their condition [21]. Studies by Saboia et al. have demonstrated that self-care applications are effective and practical for use in clinical practice as an educational technology to promote adherence to pelvic floor muscle exercises and prevent urinary incontinence in postpartum women [22].

Our study examining the effect of the self-care application on quality-of-life dimensions in women with POP found changes in four dimensions. Findings showed improvement in emotions after intervention. Consistent with this finding, Siyoum et al. demonstrated that pelvic floor muscle training significantly improved emotional responses and Personal Relationships among women with pelvic organ prolapse [23]. Our results on the severity measurement dimension showed improvement in the intervention group after two months. In line with this finding, the results of a systematic review study by Xu et al. revealed that e-health interventions are not only necessary to prevent PFD but also effective in reducing its severity. Furthermore, compared to conventional medicine, eHealth interventions have significant positive effects on several outcome measures, including quality of life, pelvic muscle strength, and sexual function. According to this research, e-health interventions are an effective treatment for PFD in women, which is consistent with our findings [24].

POP-Q stage examinations conducted before and after the intervention showed no significant differences between the groups in our study. Consistent with this finding, Wiegersma et al. reported no significant change in the degree of prolapse among women with Stage I and II POP after a three-month intervention [25]. Similarly, a study involving women with Stage III and IV POP found no change in prolapse stage after a three-month intervention [17]. In contrast, Brækken et al. demonstrated that pelvic floor muscle training combined with lifestyle advice led to improvements in both symptoms and POP-Q stage after a six-month follow-up [26]. This difference may be due to the fact that anatomical changes require a longer period of time to show improvement. The supportive structures of the pelvic floor change more slowly than psychological or behavioral components and require a longer intervention period.

Our result showed that pelvic organ prolapse–related knowledge was significantly higher in the intervention group than in the control group. This finding highlights the significant impact of educational intervention and self-care behaviors. The results of the study by Mohajeri et al. showed that approximately half of the participants had poor to moderate levels of knowledge regarding to diagnosis and treatment of PFD. Accordingly, the authors recommended that educational programs should be designed and implemented to improve women’s awareness of PFD [16]. Based on previous studies, the use of educational interventions through mobile phone programs is a useful step in this direction. The clinical trial by Saboia et al., titled “The Effect of the Continence app on the Knowledge, Attitudes, and Practices of Postpartum Women,” showed that the app-based educational intervention was effective in improving knowledge and practices related to urinary incontinence among postpartum women [22]. A 2021 qualitative study revealed that most women with pelvic floor disorders use self-care methods that they have developed through trial and error; thus, having a resource for these women can facilitate treatment [3].

### Strengths

The present study is first aimed at determining the effect of the Iranian self-care application on the quality of life of women with mild and moderate POP. In addition to pelvic floor exercises, exercise timer and reminders, this application teaches patients about POP and lifestyle recommendations. These have been suggested as important features in the design of applications for the treatment of pelvic floor disorders. Another strength of this study is that the potential for interaction between the two groups was negligible, as the activation of the application required a unique one-time authorization code. No user had any technical problems installing or using the application. Additionally, this study had no side effects.

### Limitations

Like most other research on e-health interventions, one of the limitations of our study was that participants could not be blinded to their group assignment. Additionally, the primary outcome assessor was not blinded to group allocation. However, quality of life related to POP was collected through participant self-report using standardized and validated questionnaires. Therefore, although the absence of assessor blinding may introduce the potential for procedural bias, its impact on outcome measurement is expected to be minimal. Another limitation is that the long-term effects of the application were not evaluated, and this study included a 2-month follow-up. Also, it is worth noting that this study was limited to women with mild-to-moderate prolapse, and the results may not be fully generalizable to those with more advanced stages.

## Conclusion

The results of the present study showed that a self-care application with appropriate educational content is effective in improving the quality of life and knowledge of women with symptomatic stage I-II pelvic organ prolapse. In situations where self-management is appropriate, self-care applications can be a good alternative to other treatments or can serve as a valuable supplement to other first-line treatments.

## Data Availability

All data and materials are available. Data sets used or analyzed during the current study are available upon reasonable request and without jeopardizing participant confidentiality from the corresponding author (hadizadehz@mums.ac.ir).
